# Mediation of Mucosal Immunoglobulins in Buccal Cavity of Teleost in Antibacterial Immunity

**DOI:** 10.3389/fimmu.2020.562795

**Published:** 2020-09-23

**Authors:** Hao-Yue Xu, Fen Dong, Xue Zhai, Kai-Feng Meng, Guang-Kun Han, Gao-Feng Cheng, Zheng-Ben Wu, Nan Li, Zhen Xu

**Affiliations:** ^1^Department of Aquatic Animal Medicine, College of Fisheries, Huazhong Agricultural University, Wuhan, China; ^2^Laboratory for Marine Biology and Biotechnology, Qingdao National Laboratory for Marine Science and Technology, Qingdao, China; ^3^State Key Laboratory of Freshwater Ecology and Biotechnology, Institute of Hydrobiology, Chinese Academy of Sciences, Wuhan, China

**Keywords:** evolution, mucosal immunoglobulins, B-cells, buccal mucosa, bacterial infection

## Abstract

The buccal mucosa (BM) of vertebrates is a critical mucosal barrier constantly exposed to rich and diverse pathogens from air, water, and food. While mammals are known to contain a mucosal associated lymphoid tissue (MALT) in the buccal cavity which induces B-cells and immunoglobulins (Igs) responses against bacterial pathogens, however, very little is known about the evolutionary roles of buccal MALT in immune defense. Here we developed a bath infection model that rainbow trout experimentally exposed to *Flavobacterium columnare* (*F. columnare*), which is well known as a mucosal pathogen. Using this model, we provided the first evidence for the process of bacterial invasion in the fish BM. Moreover, strong pathogen-specific IgT responses and accumulation of IgT^+^ B-cells were induced in the buccal mucus and BM of infected trout with *F. columnare*. In contrast, specific IgM responses were for the most part detected in the fish serum. More specifically, we showed that the local proliferation of IgT^+^ B-cells and production of pathogen-specific IgT within the BM upon bacterial infection. Overall, our findings represent the first demonstration that IgT is the main Ig isotype specialized for buccal immune responses against bacterial infection in a non-tetrapod species.

## Introduction

Due to its broad geographic distribution and ability to adhere to mucosal tissues, *Flavobacterium columnare* (*F. columnare*) is considered as one of the most harmful bacterial pathogens that occurs worldwide and causes columnaris disease in most freshwater fish species, including rainbow trout (*Oncorhynchus mykiss*) (1–3). The pathogen is a long Gram-negative rod in the family *Flavobacteriaceae*, one of the main phyletic lines within the *Bacteroidetes* group from the domain (4). Columnaris disease generally begins as an external infection on the skin, fins, gills, or oral cavity (5), resulting in massive mortalities and economic losses. Previous studies have showed that interaction with mucosal surfaces is critical for the pathogenesis and pathological symptoms of *F. columnare* (6, 7). Therefore, it’s necessary to understand the mechanisms responsible for mucosal immune responses to columnaris disease.

The buccal cavity (BC) represents the gateway of the gastrointestinal (GI) and respiratory tracts in vertebrates (8), and it is covered by a critical mucosal barrier (buccal mucosa, BM) that separates and protects the underlying tissues from the environment (9). Since the BM is continuously exposed to a plethora of triggers including diverse commensal microbial communities and dietary and air- or waterborne antigens that may cause infection, vertebrates have evolved an effective innate and adaptive immune system to protect the BM surface (8). In mammals, extensive and organized mucosa-associated lymphoid tissue (MALT) has been characterized in the BM (10), which contains abundant immune cells (macrophages, dendritic cells, natural killer cells, and leukocytes) and effective molecules [immunoglobulins (Igs), cytokines, chemokines, antibacterial peptides, and complement factors] (11). Interestingly, in contrast with mammals, teleost BM lacks keratinization and salivary glands (12). In addition, our previous studies have shown that diffuse MALT appears in teleost BM and contains B-cells and Igs against waterborne antigens (13).

In mammals, secretory IgA (sIgA) is produced by local plasma cells (PCs) in the stroma of the salivary glands and is then transported to the oral mucosal surface mediated by polymeric Ig receptor (pIgR) (14). Moreover, sIgA, one of the principal antibodies present in saliva, could act against cariogenic bacteria and periodontopathic bacteria as well as maintain the homeostasis of the oral microbiota by limiting the colonization of microorganisms and their invasion of the buccal epithelium (11, 15, 16). Therefore, these results indicate that sIgA is the main humoral component involved in immune responses against oral bacterial pathogens (14). Interestingly, as there are many more bacterial pathogens in water than in air, aquatic vertebrates like teleost fish must have evolved an effective mucosal immune system to protect their BC. However, the teleost fish immune response against bacterial infection in the BM remains unknown.

In contrast to mammals (mainly containing IgM, IgG, IgA, IgD, and IgE), only three Ig isotypes have been identified in teleosts (IgM, IgT/IgZ, and IgD) (17). Teleost IgM is the prevalent Ig class in serum and it appears to have strong immune responses in systemic immunity (17, 18). Although secreted IgD (sIgD) has been described coating a low percentage of the bacteria in gill and buccal mucosal surface, and a high percentage in gut of rainbow trout (*O. mykiss*) (13, 19, 20), its function remains unknown. In contrast, teleost IgT (also named IgZ) was identified in 2005 (21, 22), and it was proven to play a predominant role in the mucosal immunity of teleosts, resembling the IgA in mammals (18). Recently, the research has shown the specialization of sIgT in protection of mucosal sites from pathogens and preservation of microbiota homeostasis (23). Notably, our previous studies have shown parasite-specific IgT and IgM titers in teleost buccal mucus and serum, respectively, indicating a specialized role of IgT in BM immune responses (13). However, after bacterial infection, the local responses of mucosal B-cells and specific responses mediated by IgT in teleost BM are thus far unknown. Interestingly, bacteria-specific IgA antibodies have been detected in saliva after bacterial infection in mammalians (24, 25). Since teleost IgT and mammalian IgA have evolved through a process of convergent evolution, we hypothesize that sIgT plays a key role in fish buccal immune responses to bacterial infection. To gain evidence for this hypothesis and further insight into the evolution of buccal B-cell responses, here, we developed a bath infection model with *F. columnare* in rainbow trout, a species often used in the field of evolutionary and comparative immunology. Furthermore, severely pathological changes and pathogen loads were found in trout BM after infection. Importantly, similar to the previous studies on salivary IgA, we showed that IgT is the main buccal Ig class in responses to bacterial pathogens. Moreover, we found that the local proliferation of IgT^+^ B-cells and production of IgT occurred in trout BM resistance to bacterial infection, and thus characterized the unrecognized vital role of sIgT antibacterial infection in the BM of teleost fish.

## Materials and Methods

### Fish Maintenance

Rainbow trout (mean weight, 3–5 *g*) were obtained from a fish farm in Shiyan (Hubei, China), and maintained in the aquarium tanks (1 m × 1 m × 1 m) using a water recirculation system involving thermostatic temperature control and extensive biofiltration. Fish were acclimatized for at least 2 weeks at 16°C and fed daily with commercial trout pellets (Efico) at a rate of 0.5–1% body weight during the whole experiment periods. Animal procedures were approved by the Animal Experiment Committee of Huazhong Agricultural University.

### *F. columnare* Strain and Infection

The bacteria used in this study was *F. columnare* G_4_ strain that obtained from Professor Pin Nie’s lab in the Institute of hydrobiology Chinese academy of sciences. *F. columnare* strain G_4_ was streaked from −80°C freezer and routinely cultured in Shieh broth as described previously (7). For *F. columnare* G_4_ infection, two types of challenges were performed. In the first challenge, 60 fish (∼3–5 *g*) were challenged with *F. columnare* G_4_ via immersion at a final concentration of 1 × 10^6^ CFU ml^–1^ for 4 h at 16°C for each challenge experiment, and then transferred into the aquarium (1 m × 1 m × 1 m) containing new aquatic water. Tissue samples including BM, head kidney and spleen were collected from 6 individuals at days 1, 2, 4, 7, 14, 21, 28, and 75 after infection. Moreover, fluids (serum and buccal mucus) were taken from 12 individuals after 28 days post-infection (28 dpi, infected group). In the second challenge, another 60 fish were infected at 30 and 60 days post primary infection, and samples were taken at 75 days post-infection (75 dpi, immune group). Both experiments were performed at least three independent times. As a control (mock infected), the same number of fish were maintained in a similar tank (1 m × 1 m × 1 m) with the same culture medium without bacteria. The samples from control fish were also collected at days 1, 2, 4, 7, 14, 21, 28, and 75 post the medium culture mock challenge. Throughout this time, the fish were maintained in a flow through aquaria at 16°C, and fed daily with dry pellets at 0.5–1% biomass.

### Distribution of *F. columnare* in Trout After Infection

To observe the invasion of *F. columnare* in BM and the distribution in trout tissues, we used green fluorescent protein (GFP) labeled *F. columnare* G4 strain (with green fluorescent protein, offered by Professor Pin Nie’s lab) infected as described above. Tissue samples (BM, gill, skin, and fin) were collected at days 1, 2, and 4 after infection. On the one hand, samples were washed three times with PBS to remove the bacteria on surface. Each sample was placed into sterile sample tube and then diluted with PBS at final concentration of 0.1 *g* ml^–1^ and homogenized by TissueLyser II (Jingxin Technology) using steel beads and shaking (60 HZ for 1 min) following the manufacturer’s instructions. Then the homogenates were diluted tenfold and plated onto Shieh culture containing tetracycline, incubated at 28°C for 48 h. Bacterial counts were done in a double-blind fashion by two independent researchers using the fluorescence microscope as described previously (26). On the other hand, tissue samples (BM, gill, skin, and fin) were dissected and processed for routine histology to detect the localization of *F. columnare* used by fluorescence microscope as described previously (27). All images were acquired and analyzed using an Olympus BX53 fluorescence microscope (Olympus) and the iVision-Mac scientific imaging processing software (Olympus).

### Histology, Light Microscopy and Immunofluorescence Microscopy Studies

The tissues of rainbow trout were dissected and fixed in 4% neutral buffered formalin overnight at 4°C, embedded in paraffin, and 4 μm thick sections stained with hematoxylin and eosin (H&E) or alcian blue (AB) as described previously (13). Images were acquired in a microscope (Olympus) using the Axiovision software. For the detection of IgT^+^ and IgM^+^ B-cells, sections were double stained with polyclonal rabbit anti-trout IgT (pAb; 0.5 μg ml^–1^) and monoclonal mouse anti-trout IgM (IgG1 isotype; 1 μg ml^–1^) overnight at 4°C. After washing three times, sections were stained with Alexa Fluor 488-conjugated AffiniPure Goat Anti-Rabbit IgG (H + L) and Cy3-conjugated AffiniPure Goat Anti-Mouse IgG (H + L) (Jackson ImmunoResearch Laboratories Inc.) at 2.5 μg ml^–1^ each for 40 min at room temperature to detect IgT^+^ and IgM^+^ B-cells, respectively. For detection of pIgR^+^ cells in trout BM, we used the same methodology described previously by using polyclonal rabbit anti-pIgR antibody (pAb; 0.8 μg ml^–1^) (18). Before mounting, all sections were stained with DAPI (4’, 6-diamidino-2-phenylindole; 1 μg ml^–1^; Invitrogen). All images were acquired and analyzed using an Olympus BX53 fluorescence microscope (Olympus) and the iVision-Mac scientific imaging processing software (Olympus).

### RNA Isolation and Quantitative Real-Time PCR Analysis

Total RNA was extracted by homogenization in 1 ml TRIZol (Invitrogen) using steel beads and shaking (60 HZ for 1 min) following the manufacturer’s instructions. A spectrophotometry (NanoPhotometer NP 80 Touch) was used to quantitate the extracted RNA and agarose gel electrophoresis was used to determine the integrity of the RNA. To normalize gene expression levels equivalent amounts of the total RNA (1000 ng), each sample was used for cDNA synthesis with the SuperScript first-strand synthesis system for Quantitative PCR (qPCR; Yeasen) in a 20 μl reaction volume. The synthesized cDNA was diluted 4 times and then used as a template for qPCR analysis. The total volume of qRT-PCR amplification system were 10 μl, containing 5 μl Master mix, 0.25 μl forward primer, and 0.25 μl reverse primer (10 μM), 1 μl diluted cDNA (200 ng), and 3.5 μl nuclease-free water. The internal control gene elongation factor 1α (EF1α) was employed as reference gene. The qPCRs were performed on a 7500 qPCR system (Applied Biosystems) using the EvaGreen 2 × qPCR Master mix (Yeasen). All samples were performed the following conditions: 95°C for 5 min, followed by 40 cycles at 95°C for 10 s and at 58°C for 30 s. A dissociation protocol was carried out after thermo cycling to confirm a band of the correct size was amplified. Ct values determined for each sample were normalized against the values for housekeeping gene (EF1α). The relative expression levels of immune-related genes were shown as −ΔΔCt while the relative abundance of *F. columnare* were shown as 2^–ΔΔCt^. Primer sequences can be found in Supplementary Table S1. The relative expression level of the genes was determined using the Pfaffl’s method (28).

### DNA Extraction and PCR Amplification

To detect *F. columnare* in trout BM of different time points of experimental group, BM pieces with mucus were collected. About 10 mg BM sample was collected and homogenized by beads beating for 2 min at 60 Hz. DNA was extracted by using the E.Z.N.A.^®^ soil DNA Kit (Omega Bio-tek, Norcross, GA, United States) according to manufacturer’s protocols and assessed photometrically using a NanoDrop 2000 UV-vis spectrophotometer (Thermo Scientific, Wilmington, United States). The 16S rRNA specific primer was used to amplify the extracted DNA by thermocycler PCR system (GeneAmp 9700, ABI, United States). PCR reactions were performed in triplicate 20 μl mixture containing 4 μl of 5 × FastPfu Buffer, 2 μl of 2.5 mM dNTPs, 0.8 μl of each primer (5 μM), 0.4 μl of FastPfu Polymerase, and 10 ng of template DNA. The PCR reactions were conducted using the following program: 3 min of denaturation at 95°C, 27 cycles of 30 s at 95°C, 30 s for annealing at 55°C, and 45 s for elongation at 72°C, and a final extension at 72°C for 10 min. The PCR products were extracted from a 2% agarose gel and further purified using the AxyPrep DNA Gel Extraction Kit (Axygen Biosciences, Union City, CA, United States) and quantified using QuantiFluor^TM^-ST (Promega, United States) according to the manufacturer’s protocol.

### Proliferation of B-Cells in the BM of Trout

For proliferation of B-cells studies, we modified the methodology as previously reported by us (13, 19, 29). Briefly, control and immune fish (∼15 *g*) were anesthetized with MS-222 and intravenously injected with 200 μg EdU (Invitrogen). After 24 h, the BMs from control and survival fish were dissected, fixed and embedded in paraffin as described above. Subsequently, the paraffin sections of BM were incubated at 4°C overnight with rabbit anti-trout IgT (pAb; 0.5 μg ml^–1^) and mouse anti-trout IgM (IgG1 isotype; 1 μg ml^–1^). After washing with PBS, paraffin sections were incubated for 45 min at room temperature with Alexa Fluor 488-conjugated AffiniPure Goat anti-rabbit IgG and Cy3-conjugated AffiniPure Goat anti-mouse IgG (Jackson ImmunoResearch Laboratories Inc.) at 2.5 μg ml^–1^ each. EdU^+^ cell detection was performed according to the manufacturer’s instructions (Click-iT EdU Alexa Fluor 647 Imaging Kit, Invitrogen). Cell nuclei were stained with DAPI (1 μg ml^–1^) before mounting with fluorescent microscopy mounting solution. Images were acquired and analyzed using an Olympus BX53 fluorescence microscope (Olympus) and the iVision-Mac scientific imaging processing software (Olympus).

### Collection of Serum and Buccal Mucus

For sampling, trout were anesthetized with MS-222, and serum was collected and stored as described previously (29). To obtain the buccal mucus, we used the method described by Yu et al. (13). Fish BM tissue was excised and rinsed with PBS to remove the remaining blood. Thereafter BM tissue was incubated for 12 h at 4°C, with slightly shaking in protease inhibitor buffer (1 × PBS, containing 1 × protease inhibitor cocktail [Roche], 1 mM phenylmethylsulfonyl fluoride [Sigma]; pH 7.2) at a ratio of 250 mg of BM tissue per ml of buffer. The suspension (buccal mucus) was collected into an Eppendorf tube, and then vigorously vortexed and centrifuged at 400 *g* for 10 min at 4°C to remove trout cells. Then the cell-free supernatant was centrifuged at 10,000 *g* for 10 min at 4°C to remove the buccal bacteria from mucus. The resulting supernatant (containing buccal mucus) was harvested, filtered with 0.45 μm syringe filter (Millipore) and stored at 4°C prior to use.

### SDS-PAGE and Western Blot

Buccal mucus and serum samples were resolved on 4–15% SDS-PAGE Ready Gel (Bio-Rad) under non-reducing conditions as described previously (13, 19). For western blot analysis, the gels were transferred onto PVDF membranes (Bio-Rad). Thereafter, the membranes were blocked with 8% skim milk and incubated with anti-trout IgT (rabbit polyclone antibody, pAb), anti-trout IgM (mouse monoclonal antibody, mAb), or biotinylated anti-trout IgD (mouse mAb) antibodies followed by incubating with peroxidase-conjugated anti-rabbit, anti-mouse IgG (Invitrogen) or streptavidin (Invitrogen). Immunoreactivity was detected with an enhanced chemiluminescent reagent (Advansta) and scanned by GE Amersham Imager 600 Imaging System (GE Healthcare). The captured gel images were analyzed by ImageQuant TL software (GE Healthcare). Thereafter, the concentration of IgT, IgM, and IgD were determined by plotting the obtained signal strength values on a standard curve generated for each blot using known amounts of purified trout IgT, IgM, or IgD.

### Trout BM Explants Culture

Trout BM explants culture were used the similar method as previously described (13, 19). Briefly, control and immune fish were killed with an overdose of MS-222, and blood was removed through the caudal vein to minimize the blood content in the BM. Thereafter, approximately 20 mg of BM was submerged in 70% ethanol for 1 min to eliminate possible bacteria on their surface and then washed twice with PBS. Thereafter, tissues were placed in a 24-well plate and cultured with 400 μl DMEM medium (Invitrogen), supplemented with 10% FBS, 100 U ml^–1^ penicillin, 100 μg ml^–1^ streptomycin, 200 μg ml^–1^ amphotericin B, and 250 μg ml^–1^ gentamycin sulfate, with 5% CO_2_ at 17°C. After 7 days culture, supernatants were harvested, centrifuged and stored at 4°C prior to use the same day, otherwise, stored at −80°C until further analysis.

### Binding of Trout Immunoglobulins to *F. columnare*

To access whether infected and immune fish had generated *F. columnare*-specific immunoglobulins, we measured the capacity of IgT, IgM, and IgD from serum, buccal mucus or BM tissue explant supernatants to bind to *F. columnare* using a pull-down assay as described previously (13, 19). Initially, the *F. columnare* suspensions (1 × 10^8^ CFU ml^–1^) were preincubated with a solution of 0.5% BSA in PBS (pH 7.2) at 4°C for 2 h. Subsequently, 40 μl *F. columnare* were incubated with diluted fluids samples (buccal mucus, serum, or BM tissue explant supernatants) separately from infected, immune and control fish at 4°C for 4 h with continuous shaking in a 300 μl volume with PBS containing 1% BSA (pH 7.2). After incubation, the bacteria were washed three times with PBS and bound proteins were eluted with 2 × Laemmli Sample Buffer (Bio-Rad) and boiled for 5 min at 95°C. The eluted material was resolved on 4–15% SDS-PAGE Ready Gel (Bio-Rad) under non-reducing conditions, and the presence of IgT, IgM, or IgD was detected by western blotting using the anti-trout IgT, IgM, or IgD antibody as described above.

### Statistics

An unpaired Student’s *t*-test and one-way analysis of variance with Bonferroni correction (Prism version 6.01; GraphPad) were used for analysis of differences between groups. All data were expressed as mean ± SEM. Differences were considered significant when *P* < 0.05.

## Results

### Pathological Changes in Trout BM After Bacterial Infection

**FIGURE 1:**
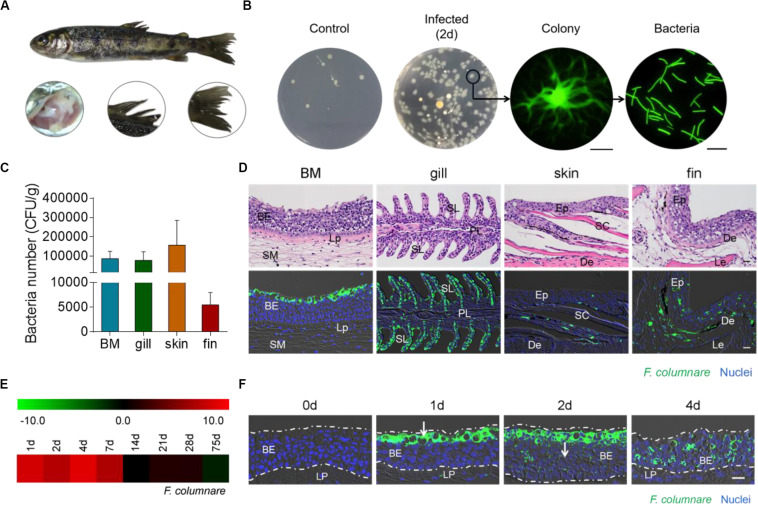
Detection and visualization of *F. columnare* in rainbow trout. **(A)** The phenotype (e.g., gill necrosis and fin rot) of rainbow trout was observed at days 2 after infection with *F. columnare*. **(B)** The culture plates from trout BM of control fish and infected fish at days 2 post-infection. Colony image: a magnified view of circled colony from infected fish by fluorescence microscope (original magnification, ×10), Scale bar, 200 μm. Bacteria image: the observation of bacterial solution obtained by circled colony expansion (original magnification, ×40), Scale bar, 10 μm. **(C)** Numbers of *F. columnare* (CFU g^–1^) was calculated by plate counting in trout BM, gill, skin, and fin tissue samples of infected fish at days 2 post-infection, respectively (*n* = 6) were 8.73 × 10^4^, 7.83 × 10^4^, 1.57 × 10^5^, and 5.51 × 10^3^ CFU g^–1^. **(D)** Histological examination by hematoxylin/eosin staining (top) and immunofluorescence staining (bottom) of trout BM, gill, skin and fin paraffinic sections from 2 days infected fish. Differential interference contrast (DIC) images showing merged staining with *F. columnare* (green) and nuclei (blue). **(E)** Heat map demonstrated results from qRT-PCR of mRNAs for *F. columnare* in infected fish versus control fish measured on days 1, 2, 4, 7, 14, 21, 28, and 75 post-infection in BM of rainbow trout (*n* = 6). **(F)** Localization of *F. columnare* in trout BM of control fish and infected fish at days 1, 2, and 4 after infection. The white arrow indicates invasion process of *F. columnare* in BM. BE, buccal epithelium; Lp, lamina propria; SM, submucosa; PL, primary lamellae; SL, secondary lamellae; Ep, epidermis; SC, scales; De, dermis; and Le, lepidotrichia. Scale bar, 20 μm. Data are representative of three independent experiments (mean ± SEM).

Here, we showed the morphological structure of trout BM containing epithelium and an underlying layer of dense connective tissue (lamina propria; Supplementary Figure S1). To observe the invasion and distribution of bacterial pathogens in trout BM, we successfully constructed a bath infection model with *F. columnare* labeled by GFP (Figure 1 and Supplementary Figure S2). At 2 days post-infection, we clearly detected that the classical phenotype of columnaris disease appeared in the trout, characterized by gill necrosis and fin rot, and we saw yellow dots suspected to be *F. columnare* on the surface of the fins and skin (Figure 1A). Interestingly, we detected a large number of bacteria with green fluorescence in trout BM from 2-day-infected fish by fluorescence microscope (Supplementary Figure S2C). Notably, the tissue homogenates of trout BM from control and 2-day-infected fish were both cultured on Shieh agar, and the bacterial colonies that were rhizoid and flat with yellow centers were detected only in infected trout. In addition, these single colonies were isolated to grow in pure culture, and characteristic elongated rod-shaped bacteria with green fluorescence were clearly observed by fluorescence microscope (Figure 1B), which further verified the successful invasion of *F. columnare* in trout BM after infection.

Next, we measured the amount of *F. columnare* by plate counting in trout BM, gill, skin, and fin tissue samples of 2-day-infected fish. Interestingly, high numbers of bacteria were detected in the BM (8.73 × 10^4^ CFU g^–1^), gill (7.83 × 10^4^ CFU g^–1^), skin (1.57 × 10^5^ CFU g^–1^), and fins (5.51 × 10^3^ CFU g^–1^; Figure 1C). Moreover, using H&E and immunofluorescence microscopy analysis, we observed the *F. columnare* mainly located on the epithelium of trout BM, gill, skin, and fins after infection (Figure 1D). By quantitative real-time PCR (qRT-PCR) and PCR, we detected the expression of *F. columnare* 16S rRNA in the BM of both trout after bacterial infection and control fish (Figure 1E and Supplementary Figure S2B). A time-series study of *F. columnare* 16S rRNA expression showed that the bacteria accumulated in trout BM mainly in the first 7 days after challenge. Importantly, we observed that *F. columnare* gathered especially on the mucus cells of the buccal epithelium on the first day after challenge, and the localization of this bacterial pathogen gradually moved down to the middle layer of the buccal epithelium over time (Figure 1F), suggesting the invasion pathway of *F. columnare* in trout BM.

### Bacterial Infection Elicits Strong Immune Responses in Trout BM

**FIGURE 2:**
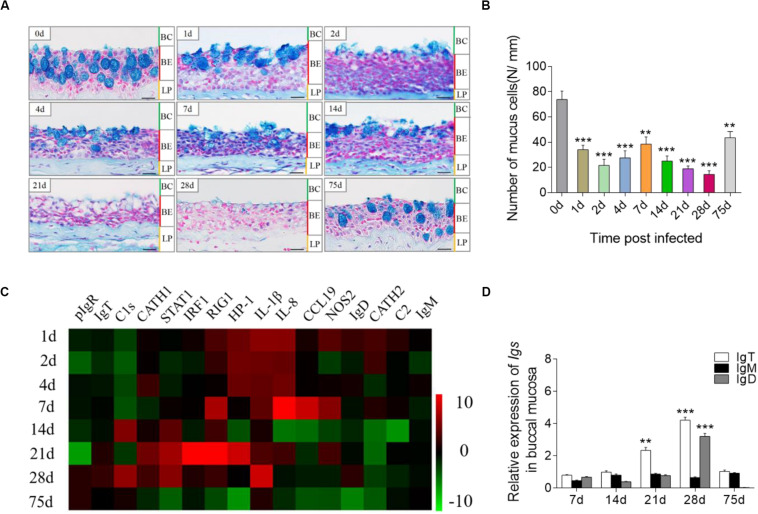
Pathological changes and immune response in the trout BM after infection with *F. columnare*. **(A)** Histological examination by AB staining of BM from trout infected with *F. columnare* after 1, 2, 4, 7, 14, 28, and 75 days and uninfected control fish (*n* = 6 fish per group). **(B)** The number of mucus cells per millimeter in the buccal epithelium of *F. columnare*-infected rainbow trout at 1, 2, 4 7, 14, 21, 28, and 75 days and control fish (*n* = 6 fish per group), counted in 25 fields from **(A)**. **(C)** Heat map illustrates results from quantitative real-time PCR of mRNAs for selected immune markers in *F. columnare*-infected fish versus control fish measured at days 1, 2, 4, 7, 14, 21, 28, and 75 post-infection (*n* = 6 per group) in the BM of rainbow trout. Data are expressed as mean fold increase in expression. **(D)** Relative expression of IgT, IgM, and IgD at days 1, 7, 28, and 75 post-infection with *F. columnare* in trout BM (*n* = 6 fish per group). BC, buccal cavity; BE, buccal epithelium; and LP, lamina propria. Scale bars, 20 μm. **P < 0.01, ***P < 0.001 (unpaired Student’s *t*-test). Data are representative of three independent experiments (mean ± SEM).

To assess the immune responses in trout BM after infection with *F. columnare*, we detected morphological changes and analyzed the expression of immune-related genes at each sampling time point. By AB staining, morphological changes were easily observed in the BM epithelium (Figure 2A), and the number of mucus cells decreased significantly at different time points post-infection, particularly at 28 days (Figure 2B). To study the mRNA expression levels of immune-related genes and cell markers in trout BM after infection, we measured 16 immune-related genes, including the cytokines [interleukin (IL) 8 and 1β], chemokine gene (chemokine-like 19), antimicrobial peptides [cathelicidin (CATH) 1 and 2], complement factors (C2 and C1s), signal transducer and activator of transcription 1 (STAT1), retinoic acid inducible gene 1 (RIG1), heterochromatin protein 1 (HP1), nitric oxide synthase 2 (NOS2), pIgR, and Ig heavy chain genes (IgT, IgM, and IgD; Figure 2C and Supplementary Figures S3A,B; primers used in this study are shown in Supplementary Table S1) by qRT-PCR. Importantly, through our studies, we characterized that strong immune responses occurred in trout BM, head kidney and spleen after challenge with *F. columnare*. Notably, in agreement with the highest level of *F. columnare* in the BM, the significantly upregulated mRNA expression of immune-related genes (e.g., CATH-2, HP1, IL-8, IL-1β, and RIG1) was detected at days 1, 2, 4, and 7 post-infection (Figure 2C). Interestingly, the expression of IgT (∼4-fold) and IgD (∼3-fold) was upregulated significantly at 28 days post-infection (Figure 2D). Moreover, in the trout head kidney and spleen, the similar expression regulation of immune-related genes was seen in the early stages of infection, while the expression of IgM was upregulated significantly at days 28 and 75 post-infection (Supplementary Figures S3A,B).

### Responses of B-Cells and Igs in Trout BM After Bacterial Infection

**FIGURE 3:**
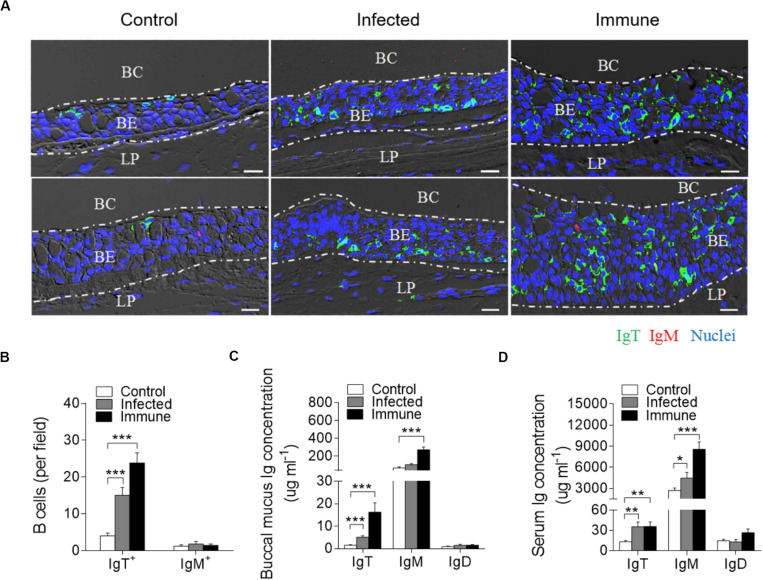
Accumulation of IgT^+^ B-cells in the BM of trout infected with *F. columnare*. **(A)** Representative DIC images of immunofluorescence staining on paraffinic sections of BM from uninfected control fish (left), infected fish (28 dpi, middle), and immune fish (75 dpi, right). IgT^+^ and IgM^+^ B-cells were stained with rabbit anti-trout IgT (green) and mouse anti-trout IgM (red), respectively; nuclei were stained with DAPI (blue; isotype-matched control antibody staining, Supplementary Figure S4 in Supporting Information). **(B)** The number of IgT^+^ and IgM^+^ B-cells in paraffinic sections of BM from uninfected control fish, infected fish, and immune fish (*n* = 6 per group), counted in 20 fields from **(A)** (original magnification, × 20). **(C,D)** Concentration of IgT, IgM, and IgD in buccal mucus **(C)** and serum **(D)** of control, infected, and immune fish (*n* = 12 per group). BC, buccal cavity; BE, buccal epithelium; and LP, lamina propria. Scale bar, 20 μm. *P < 0.05, **P < 0.01, and ***P < 0.001 (one-way ANOVA with Bonferroni correction). Data in **(B–D)** are representative of at least three independent experiments (mean ± SEM).

By immunofluorescence microscopy analysis, we observed few IgT^+^ and IgM^+^ B-cells in the buccal epithelium of control fish (Figure 3A; isotype-matched control antibodies, Supplementary Figure S4A). Interestingly, in the *F. columnare*-infected group, a moderate increase (∼4-fold) in the number of IgT^+^ B-cells was observed in the trout buccal epithelium at day 28 post-infection (Figures 3A,B). Notably, we detected substantially more accumulation (∼6-fold) of IgT^+^ B-cells on the trout buccal epithelium of immune fish (75 dpi) when compared with those of control fish (Figures 3A,B). However, the abundance of IgM^+^ B-cells did not change significantly in the infected and immune fish when compared with the controls (Figures 3A,B).

The high accumulation of IgT^+^ B-cells in trout BM after *F. columnare* challenge led us to hypothesize a critical role of IgT protein in BM. To address this hypothesis, by immunoblot analysis, we found that the IgT concentration in the buccal mucus of infected and immune fish increased by ∼3-fold and ∼11-fold when compared with control fish, respectively, which is consistent with the results of immunofluorescence. However, the concentration of IgM increased by ∼4-fold only in the immune group (Figure 3C). In serum, ∼3-fold increases of IgT concentration were detected in both infected and immune fish. Conversely, in serum, ∼ 2-, and 3-fold increases of IgM concentration were detected in infected and immune fish, respectively, when compared with control fish (Figure 3D). In contrast, the IgD protein concentration did not change significantly in either the buccal mucus or serum of the same fish groups (Figures 3C,D).

### Bacteria-Specific Ig Responses in Trout BM

**FIGURE 4:**
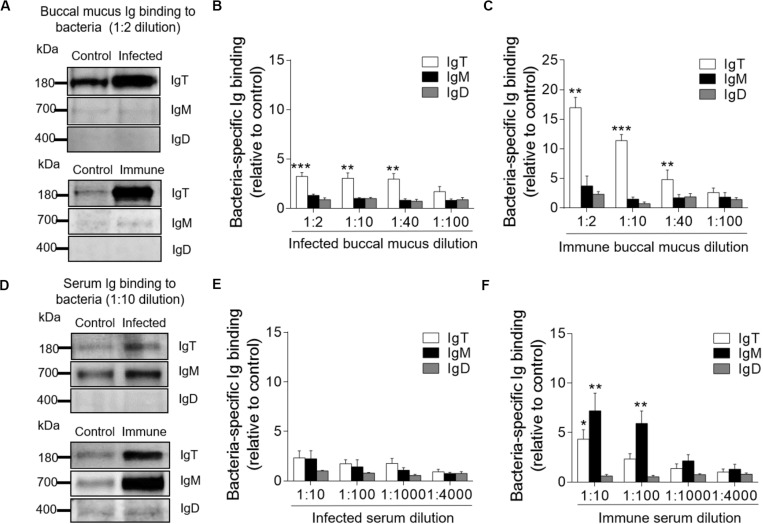
Immunoglobulin responses in the buccal mucus and serum from infected and immune trout. **(A)** Western blot analysis of IgT-, IgM-, and IgD- specific binding to *F. columnare* in buccal mucus (dilution 1:2) from infected and immune fish. **(B,C)** IgT-, IgM-, and IgD- specific binding to *F. columnare* in dilutions of buccal mucus from infected **(B)** and immune **(C)** fish, evaluated by densitometric analysis of immunoblots and presented as relative values to those of control fish (*n* = 12 per group). **(D)** Western blot analysis of IgT-, IgM-, and IgD- specific binding to *F. columnare* in serum (dilution 1:10) from infected and immune fish. **(E,F)** IgT-, IgM-, and IgD- specific binding to *F. columnare* in dilutions of serum from infected **(E)** and immune **(F)** fish, evaluated by densitometric analysis of immunoblots and presented as relative values to those of control fish (*n* = 12 per group). *P < 0.05, **P < 0.01, and ***P < 0.001 (unpaired Student *t*-test). Data are representative of three independent experiments (mean ± SEM).

The results of large increases of IgT protein levels in the buccal mucus of infected and immune fish led us to hypothesize a key role of bacteria-specific IgT in trout BM. To verify this hypothesis, we measured the capacity of Igs from buccal mucus and serum to bind to *F. columnare* with a pull-down assay (Figure 4). In buccal mucus, we detected a significant increase in bacteria-specific IgT binding in up to 1/40 dilution from both infected (∼2.9-fold) and immune fish (∼4.8-fold) when compared to control fish (Figures 4A–C). Interestingly, we did not find any bacteria-specific IgM binding in the buccal mucus (Figures 4A–C). In serum, we found a significant increase in bacteria-specific IgT binding only in the 1/10 dilution (∼4.3-fold) and only in immune fish (Figures 4D–F). In contrast, bacteria-specific IgM binding was found in up to 1/100 dilution (∼5.9-fold) of the diluted serum from immune fish (Figures 4D–F). However, bacteria-specific IgD binding was not detected in buccal mucus or serum from infected or immune fish (Figure 4).

### Local Proliferation of B-Cells and Ig Responses in Trout BM After Bacterial Infection

**FIGURE 5:**
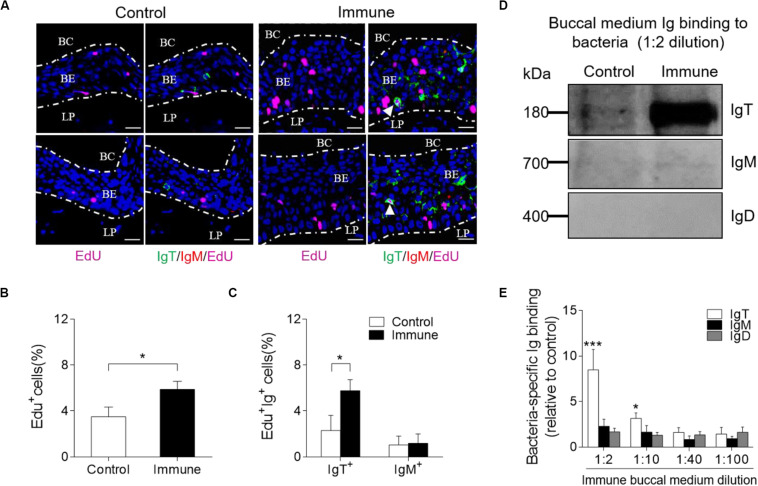
Local Igs-specific responses in BM explants and proliferative responses of IgT^+^ and IgM^+^ B-cells in the BM from immune fish. **(A)** Immunofluorescence analysis of EdU incorporation by IgT^+^ or IgM^+^ B-cells in the BM of control and immune fish. Paraffinic sections of BM were stained for EdU (magenta), trout IgT (green), trout IgM (red), and nuclei (blue) detection. White arrowheads point to cells double stained for EdU and IgT. BC, buccal cavity; BE, buccal epithelium; and LP, lamina propria. Scale bars, 20 μm. **(B)** Percentage of EdU^+^ cells from total BM cells in control or immune fish (*n* = 12 per group), counted in 20 fields from **(A)**. **(C)** Percentage of EdU^+^ cells from the total BM IgT^+^ and IgM^+^ B-cell populations in control and immune fish (*n* = 12 per group), counted in 20 fields from **(A)**. **(D)** The BM explants (∼20 mg each) from control and immune fish were cultured in medium (400 μl) for 7 days. Immunoblot analysis of IgT-, IgM-, and IgD-specific binding to *F. columnare* in the culture medium of buccal mucosa (dilution 1:2) from control and immune fish. **(E)** IgT-, IgM-, and IgD-specific binding to *F. columnare* in dilutions of buccal culture medium from control and immune fish, evaluated by densitometric analysis of immunoblots and presented as relative values to those of uninfected control fish (*n* = 12 per group). *P < 0.05 and ***P < 0.001 (unpaired Student *t*-test). Data in **(B,C,E)** are representative of at least three independent experiments (mean ± SEM).

To evaluate whether there was an increase of IgT^+^ B-cells in the trout BM of immune fish, we performed *in vivo* proliferation studies of B-cells stained with 5-Ethynyl-2’-deoxyuridine (EdU), which can incorporate into DNA during cell division. Immunofluorescence microscopy analysis showed a significant increase in the percentage of proliferating cells in the trout BM of immune fish (∼5.88 ± 0.70%) when compared with that of control animals (∼3.49 ± 0.85%; Figures 5A,B). Interestingly, we detected a significant increase in the proliferation of EdU^+^ IgT^+^ B-cells in immune fish (∼5.76 ± 0.96%) when compared with that of control fish (∼2.29 ± 1.32%; Figures 5A,C). However, no difference was detected in the percentage of EdU^+^ IgM^+^ B-cells of control fish and immune fish (Figures 5A,C). Next, we measured bacteria-specific Igs titers from the medium of cultured BM (Figures 5D,E). We detected a significant increase in bacteria-specific IgT binding in up to 1/10 diluted medium (∼3.1-fold) of cultured trout BM explants of immune fish when compared to control fish (Figures 5D,E). Interestingly, negligible bacteria-specific IgM and IgD titers were detected in the medium of cultured trout BM explants from control and immune fish (Figures 5D,E). Together, our results of the proliferation of IgT^+^ B-cells and production of IgT suggest that bacteria-specific IgT in trout BM is locally generated after bacterial challenge.

### Responses of pIgR in Trout BM After Bacterial Infection

**FIGURE 6:**
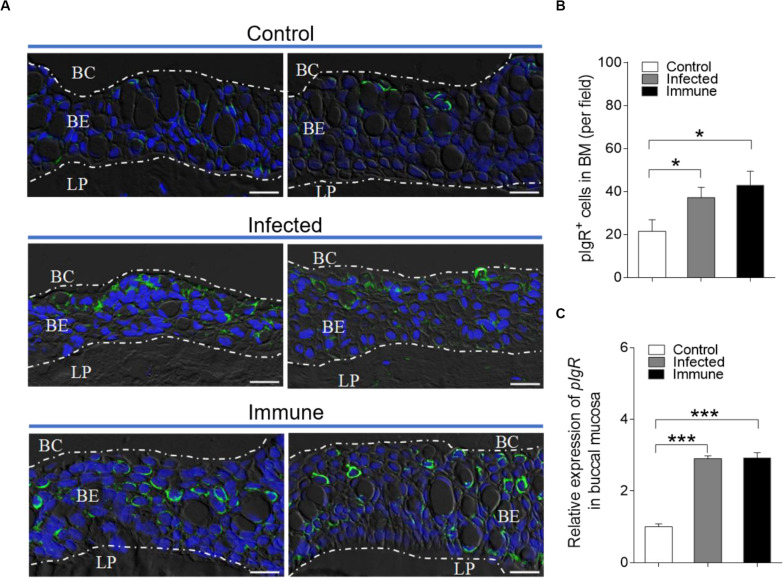
Accumulation of pIgR^+^ cells in the BM of trout after infected with *F. columnare*. **(A)** DIC images of immunofluorescence staining on trout BM paraffinic sections from control (top), infected (28 dpi, middle), and immune (75 dpi, bottom) fish, stained for pIgR (green) and nuclei with DAPI (blue; *n* = 12 fish per group; isotype-matched control antibody staining, Supplementary Figure S4 in Supporting Information). **(B)** The number of pIgR^+^ cells in trout BM paraffin sections of control, infected (28 dpi), and immune (75 dpi) fish (*n* = 12 fish per group), counted in 20 fields from **(A)**. **(C)** Relative mRNA expression of pIgR in the BM of control, infected and immune fish were detected by qRT-PCR (*n* = 6 fish per group). BC, buccal cavity; BE, buccal epithelium; and LP, lamina propria. Scale bar, 20 μm. *P < 0.05 and ***P < 0.001 (one-way ANOVA with Bonferroni correction). Data in **(B,C)** are representative of at least three independent experiments (mean ± SEM).

Our previous studies have shown that pIgR exists in trout BM and mediates the transepithelial transport of secretory Igs (13). Thus, we hypothesized that pIgR might conduct the transportation of sIgs to the buccal mucus during the immune responses to *F. columnare*. Using immunofluorescence analysis, we observed that few of the buccal epithelial cells from naïve fish were stained by the anti-trout pIgR polyclonal antibody (Figure 6A). Interestingly, a significantly increased number of pIgR^+^ cells was observed in the epidermis of trout BM from infected (28 dpi, ∼1.7-fold) and immune (75 dpi, ∼2.0-fold) fish (Figures 6A,B; isotype-matched control antibodies, Supplementary Figure S4B), respectively, when compared with those of control fish. Moreover, using qRT-PCR, the expression of pIgR was found to be significantly upregulated in the trout BM of infected (∼2.9-fold) and immune fish (∼2.9-fold), respectively, when compared with that of control fish (Figure 6C).

## Discussion

The BM is a critical first line of defense in terrestrial vertebrates (8, 11, 30). While mammals’ BC is known to contain a MALT, which plays a key role in the control of bacterial pathogens (10, 31), very little is known about the evolutionary origins of buccal MALT and its primordial roles in immune defense. Here, we report for the first time that *F. columnare* can infect the BM of rainbow trout when the fish are exposed to this type of bacteria by bath, the natural route of exposure, and elicit local mucosal immune responses in trout BM. Moreover, we show the critical role of sIgT and B-cell responses in response to *F. columnare* infection in trout BM, which indicates the evolutionary conserved functions of mucosal Igs in the BM of vertebrates.

Previous studies have shown that columnaris disease is caused by the Gram-negative bacterium *F. columnare* and severely affects the global production of many fish species (2, 32). As a mucosal bacterial pathogen, *F. columnare* infection results in the damage of mucosal tissues, with a high degree of mortality (2, 5, 32). In the present study, we successfully conducted a waterborne challenge model of rainbow trout with *F. columnare* G_4_. Notably, after 2 days of infection, clinical signs (frayed fins, depigmented lesions on the skin, and necrotic gill lesions) of columnaris disease were easily recognized, and rod-shaped bacteria that form rhizoid colonies with green fluorescence on solid growth medium could only be seen in the infected group. Moreover, by culture plate counting and histological observation, we found that the quantity and localization of *F. columnare* were different in trout BM, gills, skin, and fins, suggesting that the infection capacity and invasion rate of this bacterium vary in different mucosal tissues. These results are in agreement with the finding in mammalian BM after infection with bacterial periodontal pathogens (*Aggregatibacter actinomycetemcomitans*, *Porphyromonas gingivalis*, *Tannerella forsythia*, and *Treponema denticola*), which can induce inflammatory responses that lead to attachment loss and periodontal destruction (33, 34). Strikingly, we described that bacteria loads were highest in the BM along with the skin and similar to gill, which strongly indicates that the buccal route of infection may be one of the main targets of *F. columnare*, like skin and gill. It is interesting to note that *F. columnare* gathered especially on the mucus cells of the buccal epithelium on the first day after challenge, and the localization of this bacterial pathogen gradually moved down to the middle layer of the buccal epithelium over time. Moreover, with the occurrence of infection, significantly decreased numbers of mucus cells were observed in trout BM. These findings parallel those obtained in a former study in which the membranes of mucus cells ruptured and then the vesicle content was released, forming mucus holding antimicrobial activity and offering defense against bacterial pathogens (35–37). Overall, our results represent a unique example of a bacterial pathogen that could enter the BM of non-tetrapods.

In mammals, the buccal epithelium forms part of an intercommunicating network of the immune system, in which signals are regularly exchanged in dynamic interactions (11). However, the immune responses against bacterial pathogens in the BM of teleost fish have not yet been investigated. Here, we showed that 16 immune-related genes, including antibacterial peptides, cytokines, chemokines and complement factors, were significantly upregulated in trout BM at the early stages following *F. columnare* infection. These results correlated with the noteworthy histopathological changes in the BM of the same animals. Importantly, we found significantly increased mRNA expression levels of antimicrobial peptides, CATH-1 and CATH-2, in trout BM immediately after *F. columnare* infection, which form the first line of host defense against infectious microorganisms prior to stimulating animals’ adaptive immune systems (38–40). In addition, we described that the mRNA expression levels of proinflammatory cytokines IL-8 and IL1-β increased significantly after bacterial infection, which were induced similarly in mammal gingival epithelial cells against bacterial pathogens, *P. gingivalis* and *Lactobacillus acidophilus* (41). Combined with those of previous studies, our results indicate that *F. columnare* infection induces drastic inflammatory reactions as well as strong immune responses in the trout buccal epidermis, which is similar to what happens in the mammalian BM (10).

It has been well established that mammalian sIgA is the predominant Ig in saliva and is considered the main specific defense molecule in the BM. Notably, previous studies have shown a positive relationship between the concentration of salivary IgA and periodontal disease, which is caused by bacterial pathogens (14, 15). Interestingly, our previous report proved the presence of a diffuse MALT in trout BM, which is characterized by an epithelial layer containing a higher percentage of IgT^+^ B-cells than IgM^+^ B-cells, similar to what was previously described in other mucosa tissues of teleost (18, 19, 42–44). However, whether the IgT^+^ B-cells and sIgT play predominant roles in the BM of fish after bacterial infection is presently unknown. In this study, we found significant increases in the concentration of IgT but not IgM or IgD in the buccal mucus of infected and immune fish exposed to *F. columnare*, in accordance with the large accumulation of IgT^+^ but not IgM^+^ B-cells in the trout BM of the same individuals. Our results parallel those obtained in a previous study that indicated that trout surviving infection with the parasite *Ichthyophthirius multifiliis* exhibited large accumulations of IgT and IgT^+^ B-cells in the trout BM (13). Interestingly, similar dramatic increases of IgA secretion as well as IgA-positive cells in the salivary glands have been described in mammals following infection with the bacteria *Streptococcus mutans* (45). Interestingly, we found that the protein levels and mRNA expression of Igs were inconsistent and showed poor correlations, especially at days 75 post infection (the protein levels of IgT and IgM increased significantly while the mRNA expression not changed), this result might due to different infection methods. A constant stimulation for fish (75 dpi) made them obtain high immunity, performed as increased protein levels of Igs. However, the detection of mRNA expression was based on challenge once. Since there were many complicated and varied post-transcriptional mechanisms involved in turning mRNA into protein that were not yet sufficiently well-defined to be able to compute protein concentrations from mRNA. In addition, proteins might differ substantially in their *in vivo* half-lives (46), and these reasons caused different performance between protein and mRNA expression. Importantly, our study for the first time showed the detection of bacteria-specific titers of all three-existing teleost Igs in the buccal mucus and found pathogen-specific IgT titers mainly in the buccal mucus and to a much lesser degree in the serum of immune fish. In contrast, pathogen-specific IgM titers were detected only in the serum. Similarly, it was shown that *P. gingivalis* infection elicited *P. gingivalis*-specific IgA responses in saliva as well as IgG and IgA in serum (47). Moreover, experiments with animal models have also shown that salivary IgA specific to *Candida albicans* has an inhibitory effect on the adherence of *C. albicans* yeast cells to oral surfaces (24, 25). Overall, our results indicate that mucosal Ig (sIgT) play a key role in the BM immunity against bacterial pathogens of fish.

By *in vivo* proliferation assays, we found significant proliferative IgT^+^ B-cell responses in trout BM, suggesting that the accumulation of IgT^+^ B-cells in the BM after bacterial infection is due to local proliferation, although this remains to be fully demonstrated. In addition, the production of high titers of bacteria-specific IgT in buccal explant cultures confirmed the local production of bacteria-specific IgT and further demonstrated the presence of specific PCs in the local BM. These results parallel our previous findings on the trout gills and olfactory organ (19, 44) and suggest that trout BM acts as both an inductive and effector site of IgT responses. In contrast, mammalian BM works only as an effector site (48, 49), and most activated B-cells in the salivary glands of mammalians mainly migrate from gut-associated lymphoid tissue (GALT) and nasopharynx-associated lymphoid tissue (NALT), and the sIgA is produced by local PCs in the stroma of the salivary glands (14, 50). Hence, based on the results from previous studies in mammals and our study in trout, it suggested that although there are different molecules (sIgT versus sIgA) and cell types/glands (mucus-secreting cells versus salivary glands) in the BM of fish and mammals, functionally analogous strategies can be used to fight bacterial pathogens under evolutionary selective force.

It is well established that the transepithelial transport of secretory Igs into the mucosal surfaces is mediated by pIgR in both mammals and teleosts (17, 19, 51). Previous studies have shown that the putative trout secretory component (tSC) of pIgR is associated with sIgT in buccal mucus (13). However, the contribution of pIgR to the bacterial infection is unclear. Here, we found that trout pIgR (tpIgR) was mainly expressed in the epithelial layer of the trout BM, and we detected significantly increased numbers of pIgR^+^ cells and transcript levels of pIgR in the BM from bacterial-infected trout when compared with that in the control group. Interestingly, in a study of germ-free mice implanted with Bacteroides *thetaiotaomicron*, the expression of pIgR was upregulated (52). Thus, together with previous studies, our results strongly suggest an important role of pIgR in the transport of Igs into the buccal mucus in both mammals and teleosts after bacterial infection.

**FIGURE 7:**
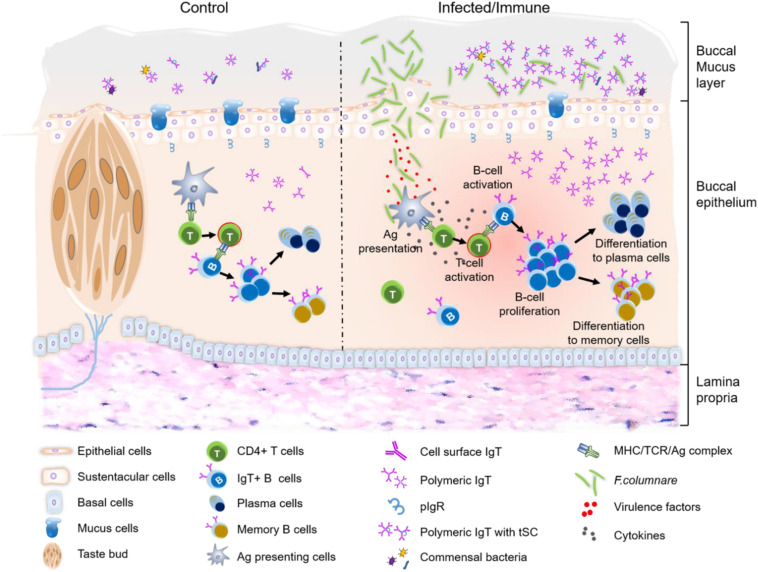
Proposed model of local IgT and IgT^+^ B-cell induction in the BM after *F. columnare* infection. The trout BM contains mucus layer, epithelium layer and lamina propria. The proposed model contains two partitions: control (left) and infected/immune (right). Induction of local IgT responses in the trout BM based on our findings. The number of IgT^+^ B-cells in control fish BM is low. IgT are produced by IgT-secreting B cells and transported from the epithelium into the mucus layer via pIgR. And the secreted IgT coats the majority of commensal bacteria in the BM surface (5). When *F. columnare* invaded the BM, antigen (Ag) can be taken up by antigen-presenting cells (APC) and presented to naive CD4^+^ T-cells. Ag-specific CD4^+^ T-cells then produce cytokines to activate B cells. Activated B cells start proliferating and may differentiation to plasma cells to locally produce *F. columnare*-specific IgT, which will be transported by pIgR into buccal mucus where can specially binding to the *F. columnare*. Alternatively, some IgT^+^ plasma cells may differentiate into memory IgT^+^ B-cells. When *F. columnare* invade the host again, the memory IgT^+^ B-cells would directly proliferate and differentiate into plasma cells, and then rapidly produce specific-IgT to bind *F. columnare*.

In conclusion, our results provide the first evidence for the process of bacterial invasion in trout BM. Moreover, following bacterial infection, pathological changes, immune-related gene upregulation, as well as B-cell proliferation and bacteria-specific IgT production events occur within trout BM (Figure 7). Thus, from an evolutionary viewpoint, our results not only expand our view of buccal immune systems from a novel perspective but also reinforce the idea of mucosal Igs (sIgT and sIgA) specialized in fish and mammalian mucosal immunity through primordially conserved principles. In addition, since many pathogens invade fish through the BM, our findings suggest that buccal vaccination may be an effective way to prevent aquatic bacterial diseases.

## Data Availability Statement

The original contributions presented in the study are included in the article/Supplementary Material, further inquiries can be directed to the corresponding author.

## Ethics Statement

The animal study was reviewed and approved by The Animal Experiment Committee of Huazhong Agricultural University.

## Author Contributions

ZX designed the research. H-YX and FD performed most of the experiments and contributed to conduct the infection model. XZ and G-KH contributed to the immunofluorescence analysis. K-FM contributed to western blot analysis. G-FC and Z-BW contributed to real-time analysis. H-YX, FD, NL, and ZX wrote the manuscript. All authors contributed to the article and approved the submitted version.

## Conflict of Interest

The authors declare that the research was conducted in the absence of any commercial or financial relationships that could be construed as a potential conflict of interest. The reviewer, FT, declared a past co-authorship with one of the author, ZX, to the handling editor.

## Supplementary Material

Supplementary Figure 1 An overview of rainbow trout buccal area. **(A)** The image representing the anatomical location and morphology of trout buccal area. **(B,C)** Histological examination by Hematoxylin/eosin (H&E) staining of trout BM **(B)** and enlarged images **(C)** of the areas outlined. BE, buccal epithelium; LP, lamina propria; and SM, submucosa. Scale bar, 20 μm.

Supplementary Figure 2 Successful infection with *F. columnare* in BM of trout. **(A)** Percentage survival of control and fish infected with *F. columnare* (*n* = 60 fish per group). **(B)** Detection of *F. columnare* by PCR in trout BM at days 1, 2, 4, 7, 14, 21, 28, and 75 after infection. **(C)** Representative DIC images of trout BM tissues isolated from infected fish at days 2 post-infection. The respective images were obtained and merged as illustrated. Scale bars, 100 μm. *P* < 0.01 (unpaired Student’s *t*-test). Data are representative of three independent experiments (mean ± SEM).

Supplementary Figure 3 Kinetics of the immune response in head kidney and spleen of trout after infected with *F. columnare*. **(A,B)** Heat map illustrates results from qRT-PCR of mRNAs for selected immune markers in *F. columnare*-infected fish versus control fish measured at days 1, 2, 4, 7, 14, 21, 28, and 75 post-infection in trout head kidney **(A)** and spleen (**B**; *n* = 6 fish per group) Data are representative of three different independent experiments (mean ± SEM).

Supplementary Figure 4 Isotype control staining for anti-IgT, anti-IgM, and anti-pIgR antibodies in trout BM paraffin-sections. DIC images of buccal paraffin-sections from control fish, with merged staining of isotype control antibodies for anti-trout IgT pAb (green) and anti-trout IgM (red) mAb **(A)**, or anti-trout pIgR (green) mAb **(B)**. Nuclei were stained with DAPI (blue). BC, buccal cavity; BE, buccal epithelium; LP, lamina propria. Scale bars, 20 μm. Data are representative of three independent experiments.

Supplementary Table 1 Primers used in this study.
