# Supplementary material for: Mediation of Mucosal Immunoglobulins in Buccal Cavity of Teleost in Antibacterial Immunity
Source: Front Immunol. 2020 Sep 23;11:562795. doi: 10.3389/fimmu.2020.562795 (PMC7539626; doi:10.3389/fimmu.2020.562795)
Supplement: Supplementary Figure 1 — An overview of rainbow trout buccal area. (A) The image representing the anatomical location and morphology of trout buccal area. (B,C) Histological examination by Hematoxylin/eosin (H&E) staining of trout BM (B) and enlarged images (C) of the areas outlined. BE, buccal epithelium; LP, lamina propria; and SM, submucosa. Scale bar, 20 μm. [file Image_1.pdf]

Supplementary materials

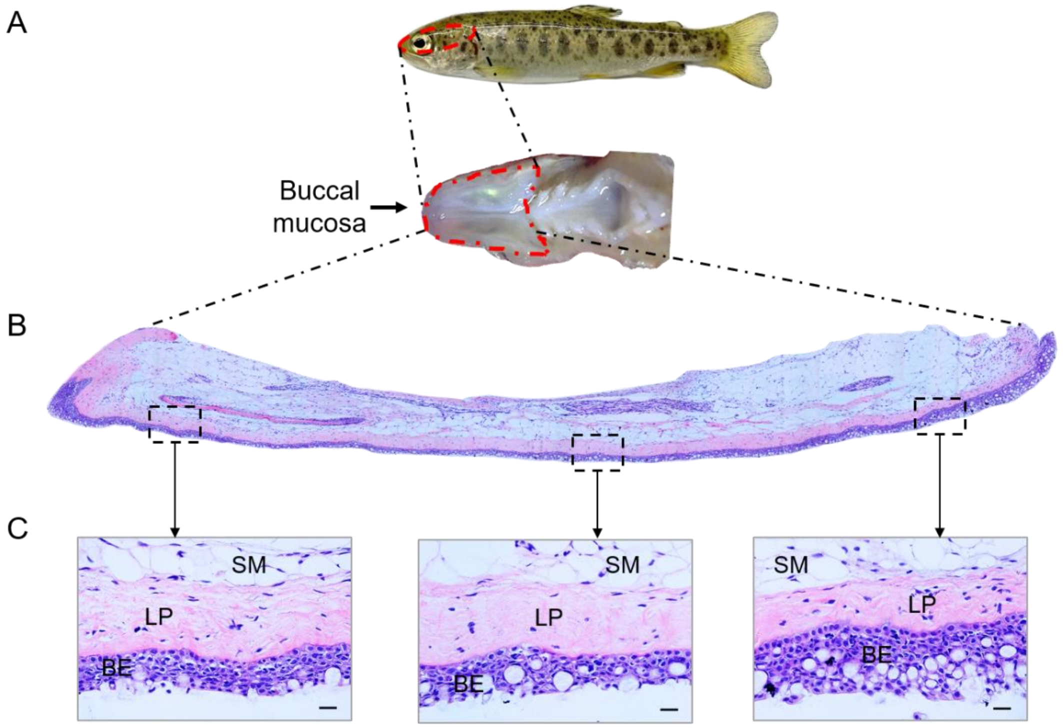

FIGURE S1 | An overview of rainbow trout buccal area. **(A)** The image representing the anatomical location and morphology of trout buccal area. (**B, C**) Histological examination by Hematoxylin / eosin (H&E) staining of trout BM (**B**) and enlarged images (**C**) of the areas outlined. BE, buccal epithelium; LP, lamina propria; SM, submucosa. Scale bar, 20 μm.

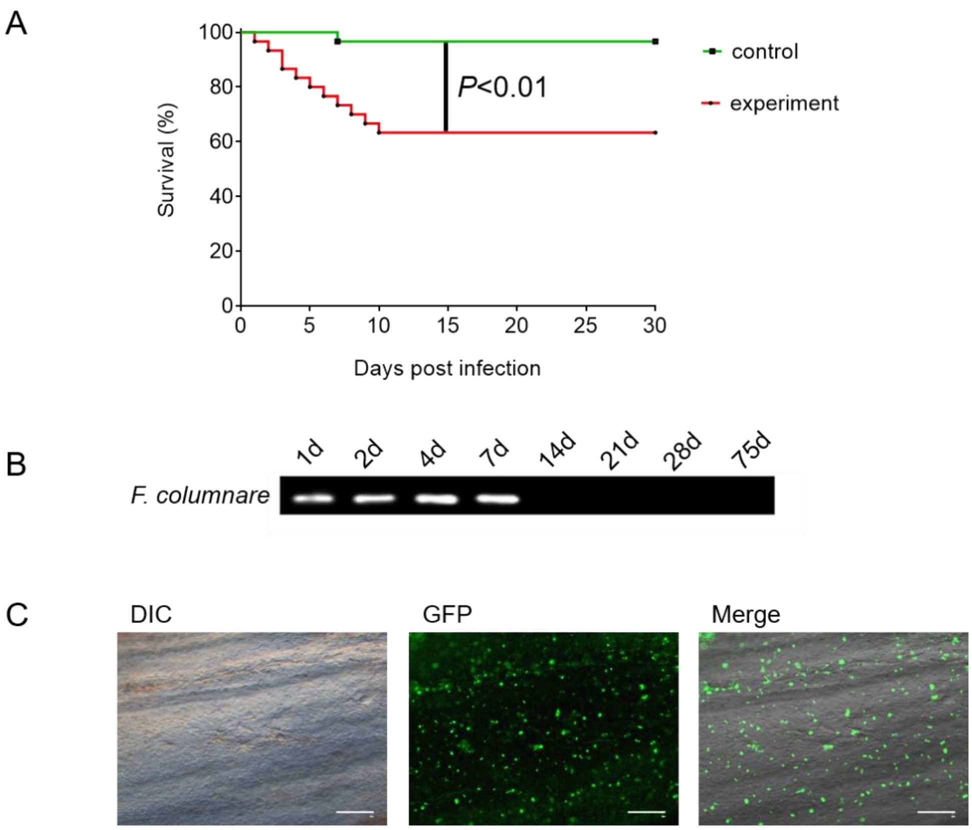

FIGURE S2 | Successful infection with *F. columnare* in BM of trout. **(A)** Percentage survival of control and fish infected with *F. columnare* (*n* = 60 fish per group). **(B)** Detection of *F. columnare* by PCR in trout BM at days 1, 2, 4, 7, 14, 21, 28 and 75 after infection. **(C)** Representative DIC images of trout BM tissues isolated from infected fish at days 2 post-infection. The respective images were obtained and merged as illustrated. Scale bars, 100 µm. $P < 0.01$ (unpaired Student's *t*-test). Data are representative of three independent experiments (mean ± SEM).

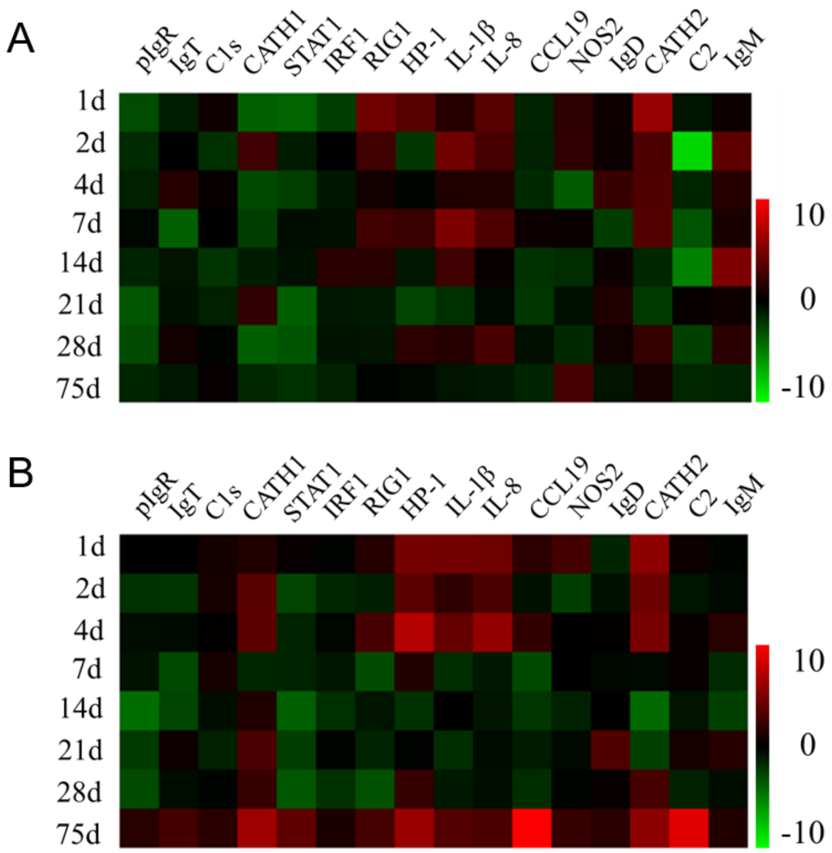

FIGURE S3 | Kinetics of the immune response in head kidney and spleen of trout after infected with *F. columnare*. **(A, B)** Heat map illustrates results from qRT-PCR of mRNAs for selected immune markers in *F. columnare*-infected fish versus control fish measured at days 1, 2, 4, 7, 14, 21, 28 and 75 post-infection in trout head kidney **(A)** and spleen **(B)** (*n* = 6 fish per group) Data are representative of three different independent experiments (mean ± SEM).

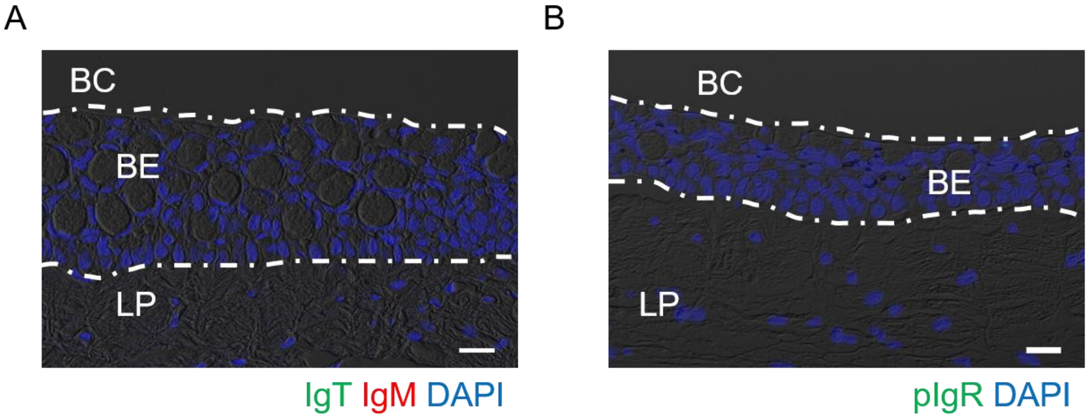

FIGURE S4 | Isotype control staining for anti-IgT, anti-IgM and anti-pIgR antibodies in trout BM paraffin-sections. DIC images of buccal paraffin-sections from control fish, with merged staining of isotype control antibodies for anti-trout IgT pAb (green) and anti-trout IgM (red) mAb **(A)**, or anti-trout pIgR (green) mAb **(B)**. Nuclei were stained with DAPI (blue). BC, buccal cavity; BE, buccal epithelium; LP, lamina propria. Scale bars, 20 µm. Data are representative of three independent experiments.

TABLE S1 | Primers used in this study.

Gene	GenBank accession no.	Primer Sequence (5' – 3')	
		Forward primer	Reverse primer
16S rRNA	EU395796.1	GAGTGGCTAAGCGAAAGTGAT	ACCTGACACCTCACGGCAC
IgD	JN173049.1	CAGGAGGAAAGTTCGGCATCA	CCTCAAGGAGCTCTGGTTTGGA
pIgR	FJ940682.1	AGAAGCGTTGGTGTCGTA	AAGCCTTGGTCAGGTCAT
IgM	OMU04616	AAGAAAGCCTACAAGAGGGAGA	CGTCAACAAGCCAAGCCACTA
IgT	AY870264	CAGACAACAGCACCTCACCTA	GAGTCAATAAGAAGACACAACGA
STAT1	NP_001118179.1	GACCAGCGAACCCAAGAACCTGAA	CACAAAGCCCAGGATGCAACCAT
EF-1α	XM_021571866.1	CAACGATATCCGTCGTGGCA	ACAGCGAAACGACCAAGAGG
CATH-1	NM_001124480.1	CTGGAGGCAAGCAACAAC	CCCCCAAGACGAGAGACA
IL-1β	NM_001124347.2	TGATGAATGAGGCTATGGA	GATGGTGAAGGTGGTAAGG
IRF1	NP_001239293.1	CGAGACTACACCAGACCCTA	TTGCTTTTGACCTCTTGTTATT
NOS2	XM_021581479.1	GGCAGTCAAGAACCAACC	GAGCACCAAACGCTAATT
HP-1	XM_021607150.1	CGGAGGAGGTTGGAAGC	GCAGCAGAAGCCACAGC
C1s	XM_021581979.1	AACAAGCCAATGGTTTTCAC	GATTCCTTTCCCAGTTCACA
IL-8	NM_001124362.1	TGTCGTTGTGCTCCTGG	CCTGACCGCTCTTGCTC
C2	NP_001117673.1	CAGCATCTCCAAGACCCACT	TCCTTACGTTGCTCCTGACA
RIG1	AGN48009.1	CAGAGGTACTACAGGAAATGG	TTACTGGTCTTCAAGCAATG
CATH-2	AY542963	ACATGGAGGCAGAAGTTCAGAAGA	GAGCCAAACCCAGGACGAGA
CCL19	XM_021602563.1	GCTGCCACTGTGTTTGTC	CTGTCCTTTCCCTTATGC
